# The importance of analyzing neighbor competitive response in the target–neighbor experimental design

**DOI:** 10.1002/ece3.1689

**Published:** 2015-10-26

**Authors:** Kevin J. Barry, Michele R. Dudash

**Affiliations:** ^1^ Department of Biology University of Maryland College Park Maryland 20742; ^2^ Department of Biology West Virginia University Morgantown West Virginia 26506

**Keywords:** Competitive response, experimental controls, experimental design, plant competition, target–neighbor

## Abstract

The role of competition in community structure and species interactions is universal. However, how one quantifies the outcome of competitive interactions is frequently debated. Here, we review the strengths and weaknesses of the target–neighbor design, a type of additive design where one of the competing species is reduced to a single individual and where controls and analyses are used for the target, but not for the neighbors. We conducted a literature review to determine how the target–neighbor design has been typically used and analyzed. We found that historically, targets were often smaller than neighbors and introduced after neighbor establishment; thus, targets would have little effect on neighbors. However, as co‐establishment of targets and neighbors of similar size is now common, the target is more likely to affect the neighbors than in its earlier usage. This can be problematic, because if targets have a significant effect on neighbor performance, bias is introduced into the assessment of the target results. As target treatment controls are necessary to determine the absolute effect of neighbors on target growth, we advocate that analysis of the neighbor competitive response serves as a necessary control for unexpected target x neighbor interactions.

## Introduction

The study of competition among species has led to the development of many concepts and theories of species coexistence (Darwin 1859; Paine 1974; Harper 1977; Sax et al. 2005). Among plants in particular, there is a long history of empirical experiments designed to examine inter‐ and intraspecific interactions (Clements et al. 1929; Connell 1983). Knowledge of plant interactions is essential for understanding species' distributions (Pellissier et al. 2010), succession (Tognetti et al. 2010), evolution (Darwin 1859; Pfennig and Pfennig 2010), as well as the spread of invasive species (Holdredge and Bertness 2011; Murrel et al. 2011). A thorough understanding of interspecific interactions is especially important as climate change alters interactions between plant species within a community (Dunnet and Grime 1999; Adler et al. 2009) and often appears to benefit invasive species (Bradley et al. 2010; Willis et al. 2010). Although there has been much research into the mechanisms of plant competition, the role of competition in structuring plant communities is still a source of debate (e.g., Went 1973; Craine 2005).

A variety of experimental designs have been utilized to address a myriad of questions involving plant competition. The advantages and drawbacks to these designs are well documented (Gibson et al. 1999) and frequently debated (Cousens 1991; Gibson et al. 1999; Freckleton et al. 2009). The majority of the experimental designs still in use have not changed substantially since their original introduction (Gibson et al. 1999), though some, such as the replacement series, has lost favor to designs with fewer confounding factors. The optimal analyses for the data obtained from each of these experiments are also debated, as are the most informative metrics of competition that can be derived from the data (Freckleton and Watkinson 2000; Weigelt and Jolliffe 2003; Freckleton et al. 2009; Onofri et al. 2010).

Manipulative plant competition experiments typically contain some form of control treatment. These control treatments serve as a comparison to the plants in competition and most often take the form of either a single plant grown without competitors or as a monoculture of the species of interest. One particular plant competition experimental design, the target–neighbor design, is unique in this aspect in that one of the experimental groups, the “neighbors,” is not controlled for in a separate treatment. (Gibson et al. 1999). The target–neighbor design is a form of additive design where one of the competing species is represented by a single individual (the target) and only the density or identity of the surrounding individuals (the neighbors) is manipulated (Goldberg and Fleetwood 1987). This design is advantageous because it focuses on the response of an individual plant, rather than the mean of a population (Gibson et al. 1999). Although density and proportions can be confounded in certain circumstances, this is not an issue when neighbor density is held constant, and only neighbor identity is manipulated as a treatment.

Within a given target–neighbor design experimental plot, each plant belongs to one of the two groups: target or neighbors. The outcome of the experiment is then dependent on the interactions between these two groups, and their respective competitive effects and competitive responses (Goldberg and Landa 1991). The *competitive effect* of an individual is its influence on its competitors, while the *competitive response* is its reaction to the presence of those competitors (Panetta and Randall 1993). In a system where only two individuals or groups (e.g., target and neighbor) are planted in competition with one another, the competitive effect of one group is equal to the competitive response of the other group. However, in a target–neighbor experiment with two or more different targets and two or more neighbor treatments, the competitive effects and competitive responses of both the target individuals and the neighbor communities are distinct from one another. The competitive effect of a neighbor treatment is its influence *on* the targets. The competitive response of a neighbor treatment is the neighbors' *reaction to* the targets. Likewise, target competitive effect is the influence of that target on its neighbors, and target competitive response is the target's reaction to the neighbors. The respective competitive effect and competitive response of a species may even be associated with specific traits (Wang et al. 2010) and vary across those traits, indicating the importance of understanding both of these aspects of competition within an experiment.

One of the earliest appearances of the target–neighbor design was in the chapter “Competition in Underplanted Cultures” in the book Plant Competition by Clements et al. (1929). To separate the effects of competition for light and water, *Helianthus annuus* cultures were planted at various densities in a pot with a separate cylinder sunk in the center. Once the neighbors reached a certain size, seed was sown in the center cylinder. The central plant (sometimes multiple plants) served as a phytometer that grew beneath larger conspecifics. Following this precedent, the target–neighbor design was traditionally used for studies focusing on separation of above‐ and belowground competition (McPhee and Aarssen 2001).

More recently, however, the target–neighbor design has been used less frequently for separation of above‐ and belowground studies and is now used almost solely in experiments where targets and neighbors are simultaneously grown together, neighbor density or identity is manipulated, and only aboveground performance is assessed. It is this shift in target–neighbor design usage and the potential for experimental bias due to the effect of target growth on neighbor performance that warrants a reexamination of the analyses that are used to assess the competitive effects of both targets and neighbors. To begin, we first searched the literature to establish past and current precedents and then proposed a means of controlling for unexpected bias through specific measurements and analyses of the neighbor treatments.

## Review of Target–Neighbor Usage

We conducted a literature review of peer‐reviewed ecological journals in order to determine how target–neighbor experiments were utilized and how their results were analyzed. We searched ISI Web of Science using the search terms “target neighbor” or “target neighbour” (American and British spellings, respectively) and “plant,” with no restriction on year through June 2014. From these results, we selected manipulative studies where targets were deliberately planted into a neighbor community consisting of at least two neighboring individuals. We omitted those studies where targets were planted with only one neighbor, as these are more accurately defined as pairwise designs. Studies were then divided into those where the experiment was entirely manipulative, with all plants deliberately planted in a target–neighbor design, and those where the target was planted into a natural or unstructured (i.e., seeds were randomly sowed) neighbor community. This was done because when targets are transplanted into a natural or unstructured community, neighbor identity, number of neighbors, and position of the target relative to its neighbors are a challenge to control across replicates and consistent neighbor measurements would not be feasible in this situation. Additionally, we noted whether the experiment separated aboveground performance from belowground performance to assess competition.

Those studies that were entirely manipulative were then characterized based on four points: (1) whether either the neighbor competitive effect or target competitive response was reported (both are the measures of neighbor influence on the target), (2) whether more than one target treatment was used, (3) whether neighbor competitive response was reported, and (4) whether the targets and neighbors were planted simultaneously (Appendix S1).

We found a total of 71 published studies that used the target–neighbor design. We focused our review on the 49 studies that met our criteria of a manipulative target–neighbor design where both the target and neighbors were deliberately planted. All but one of these studies reported a neighbor competitive effect or target competitive response*,* as is expected with the target–neighbor design. Only 25 of 49 studies (51%) used at least two different target treatments per neighbor treatment in their experimental designs and could have potentially analyzed a separate neighbor *competitive response*. We focused on these multiple target studies because where only one target treatment is used, the target competitive effect and neighbor competitive response are one in the same. Of these 25 studies where a separate neighbor competitive response could potentially be measured, only one reported a neighbor competitive response, where they found a significant target × neighbor treatment interaction (Cheplick and Kane 2004). One other article (Thijs et al. 1994) reported a neighbor response to an herbicide treatment but not to the target treatments. Our review of the literature demonstrates that in the common usage of the target–neighbor design, the influence of the neighbors on the target was analyzed, but an analysis of the influence of the *target on the neighbors*, specifically the neighbor competitive response, was almost universally unreported.

## Target–Neighbor Design – Past and Present

Early usages of the target–neighbor design were in aboveground and belowground separation studies which usually relied on planting relatively small target seedlings into pre‐established communities of neighbors (Appendix S1). In these cases, one would expect little effect of the target on the neighbors (Cook and Ratcliff 1984; Goldberg and Fleetwood 1987). Clements et al. (1929) reported only the competitive effects of the treatments on the phytometers (targets) but not on the neighbors. This was reasonable as the target seedling would have little to no measureable effect on the growth of the larger established neighbors. Welbank (1961) early on recognized that focusing only on target responses and not neighbor responses was an incomplete analysis of their competitive interactions. However, Welbank specified that this incomplete approach was justified in order to simplify the experiment, as it was especially practical for studies of crop–weed competition where crop effects on weeds would not be interesting. Likewise, the smaller target crops used in this study would not be as likely to affect the larger weed neighbors' growth (Welbank 1961).

Prior to 2000, 13 of 35 target–neighbor studies had co‐established the targets and neighbors, compared to 25 of 29 studies since 2000 (Appendix S1). Several authors explicitly stated that targets and neighbors were co‐established in order to eliminate size biases in the examination of competition (e.g., Hwang and Lauenroth 2008). This practice increases the probability that the targets and neighbors will be similar in size, thus increasing the probability that targets will have a significant effect on neighbor growth. Therefore, analysis of neighbor competitive response was more relevant than in earlier studies when targets were smaller or planted into an established neighbor community (e.g., Cook and Ratcliff 1984). Despite the increased probability that targets will influence neighbor characteristics, however, the analyses of performance and the results presented from target–neighbor experiments have remained largely unchanged since the experiment's first appearance in the literature.

Analysis of neighbor competitive response provides greater insight into interactions between targets and neighbors through determination of whether neighbor community characteristics vary across different target treatments. In a target–neighbor experiment with two or more separate target treatments, valuable information about the treatments could be missed without this analysis. In an experiment where the independent variable is an abiotic factor, such as a fertilizer treatment, it would be expected that each treatment that was to receive a given amount of fertilizer would indeed receive that amount. Obviously, no difference in fertilizer application within replicates of the same treatment should be expected. Alternatively, in any experimental design where plants are manipulated as a treatment, some variation is unavoidably present in that treatment variable. If this variation becomes so great that there is a statistical difference within a neighbor treatment when it is planted with separate target treatments, the growth of the neighbors will be dependent on the growth of their respective targets, and the characteristics of the neighbor communities will vary along with the target. In this case, bias can be introduced, as the neighbor treatments are no longer uniform across targets.

One could take the view that as long as the neighbor treatments were uniform at the start of the experiment, then any differences that develop within the competitive response of the neighbor treatment are an acceptable part of the interactions between target and neighbor plants and thus do not need to be accounted for. We suggest that a broader, more inclusive view of experimental interactions should be taken. Whether or not the neighbor competitive response is of explicit interest, there should be recognition that a neighbor treatment can vary across targets and that if this neighbor treatment variation is not accounted for, the conclusions made from comparisons of competitive interactions among different targets grown within the same neighbor treatment may themselves be biased. A narrow view is more restrictive toward species interactions that are not of interest, but a lack of interest in those interactions does not mean that those interactions, which are easily measured, are not relevant to the study.

**Figure 1:**
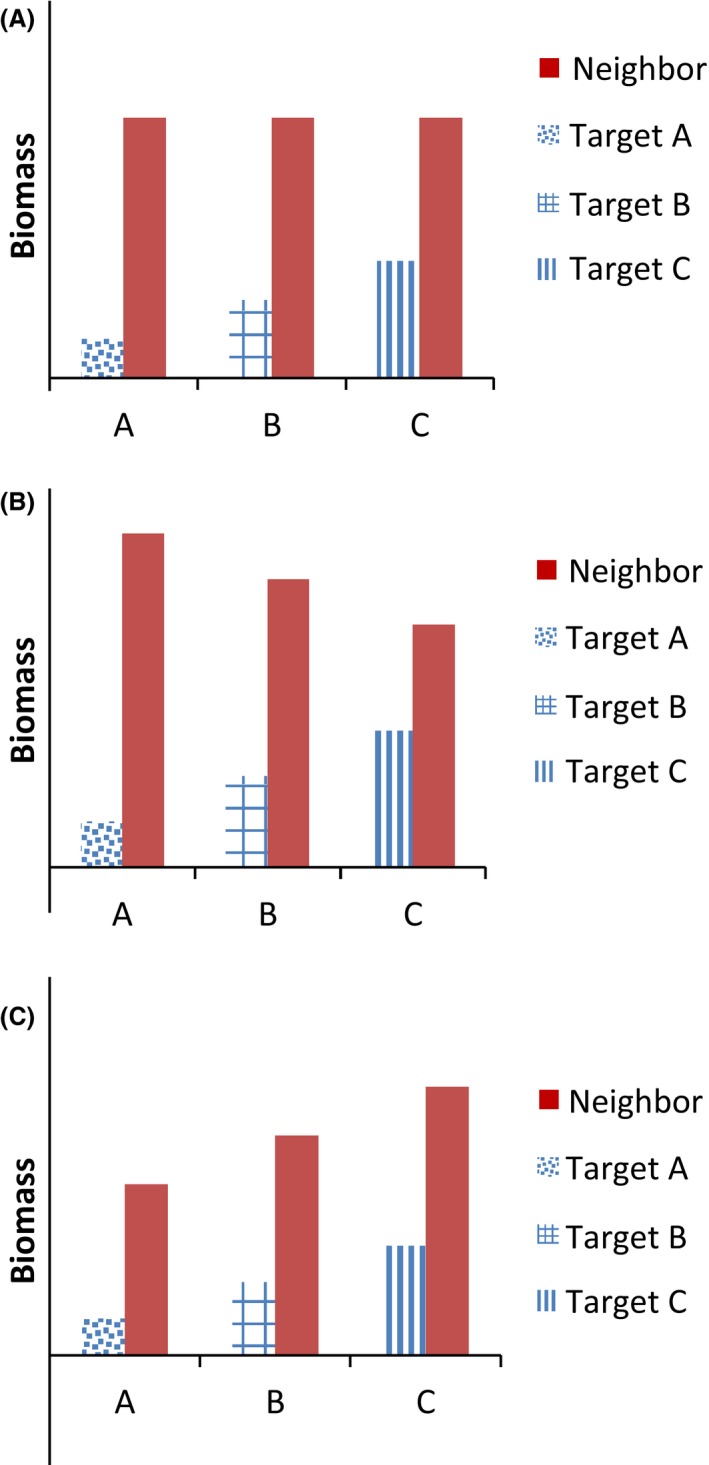
Graphic illustrating three potentially different competitive outcomes between the targets and neighbors of the target–neighbor experimental design when grown together simultaneously. The response variable above is ground biomass. The neighbor treatments (blue bars) are shown with the three target treatments (red bars) in three possible scenarios: (A) Neighbors are not differentially affected by targets (no difference in neighbor competitive response). (B) Neighbors are differentially affected in a negative fashion by target biomass (competition). (C) Neighbors are differentially affected in a positive fashion by target biomass (facilitation).

**Figure 2:**
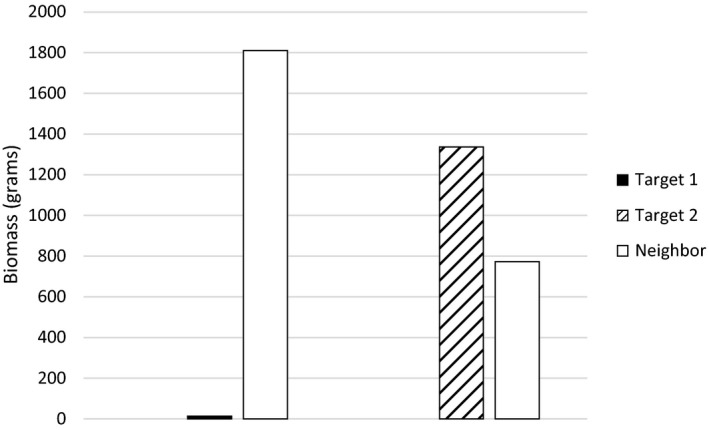
Vegetative biomass competitive response means for two different targets and for their associated late phenology neighbor treatments when grown together. Data taken from K. Barry 2013.

To illustrate the importance of a neighbor competitive response, we diagram three scenarios to illustrate the potential outcomes (Fig. 1) and then provide empirical data from our experimental work. In our empirical data, there was a significant difference in the biomass of a neighbor treatment when grown with two separate targets, which could have potentially altered the interpretation of the target competitive response results (Fig. 2, modified from K. Barry Dissertation, 2013).

When neighbor competitive response is not reported, the implicit assumption is that neighbor characteristics such as height or biomass are equivalent across all target treatments (Fig. 1A). If this first scenario is true, then the targets would have no significant effect on neighbors, and neighbor characteristics would not vary in response to the targets. This also means that any variation in overall treatment plot characteristics, such as plot biomass, is dependent solely on variation among the different target species. There are several nonmutually exclusive reasons for this result. For instance, if neighbors were established prior to the targets, resource preemption by the neighbors could result in only the targets experiencing a reduction in growth (Craine et al. 2005). Alternatively, targets may be considerably smaller than neighbors, or targets and neighbors could have different resource requirements, causing the target to have little influence on neighbor growth.

In the second scenario, if targets are planted simultaneously with the neighbors, are of a similar size, or even larger than the neighbors, it is more likely that there would be a negative effect of a target on the neighbors and that variation in target size would lead to differences in neighbor competitive response (Fig 1B). Although intensity of competitive effect and response are not necessarily correlated (Wang et al. 2010), plants that have strong competitive effect on neighboring individuals often have a limited competitive response. A target with these characteristics, or a target that is a stronger competitor than the neighbors, would also be likely to influence neighbor characteristics. This pattern may also imply that maximum plot biomass is restricted by limited resource availability (Clements et al. 1929; Harper 1977). In this case, an increase in target biomass would lead to a proportional decrease in neighbor biomass.

In the third scenario, there can be facilitation between target and neighbors (e.g., through nitrogen fixation or soil moisture retention by one of the partners) resulting in a positive relationship between target and neighbor biomass (Fig. 1C). Brooker et al. (2008) suggest that facilitation is an understudied phenomenon that should be measured in more than just extreme environments. Although it is more likely for the neighbors to facilitate the target than vice versa because there are multiple neighbors surrounding the single target, actual target–neighbor relationships would be dependent on the relative sizes and characteristics of the targets and the neighbors (K. Barry Dissertation, 2013).

In an empirical study to examine target–neighbor competitive effects, we observed a variable neighbor competitive response when two different targets were planted with the same neighbors (K. Barry Dissertation, 2013). Here, all targets and neighbors were planted simultaneously, as is done in most current target–neighbor studies. There was a statistically significant difference in neighbor treatment biomass (Fig. 2), but not in other variables that we measured, including fruit mass and flowering time. This result corresponds to the scenario in Figure 1B. The significant difference in neighbor treatment biomass when grown with the two targets introduced a bias into the neighbor treatment, because that treatment is no longer consistent.

## Target–Neighbor Design – Solutions

To detect a significant difference in neighbor competitive response, neighbors must first be measured. Although the targets were always measured in the target–neighbor designs we reviewed, this was not the case for the neighbor plants. Expectedly, the experimental hypotheses were related to target performance in different neighbor communities, but an interest solely on target performance, and not in the neighbors, does not mean that the neighbor treatments were free of bias and thus affecting target results.

A difference in neighbor competitive response is indicated by a significant effect of target treatment on neighbor characteristics, or a significant target × neighbor interaction when analyzing neighbor characteristics (Fig. 1B). In the single study that reported a neighbor competitive effect (Cheplick and Kane 2004), the objective was to analyze the growth of targets grown with neighbors that belonged to either the same or different maternal families. Unlike most target–neighbor studies, they were *not* comparing target performance across the different neighbor treatments, and it was therefore not necessary to qualify their conclusions as a result of the target–neighbor interaction. The vast majority of studies were interested in target performance across the different neighbor treatments, however.

There are multiple approaches for dealing with this bias. One solution to this problem is to use the variable neighbor trait as a predictor or covariate during analysis (e.g., Howard 2001; Weigelt et al. 2002). If many traits vary within the neighbor treatment, for example, both neighbor biomass and neighbor phenology, each of these characteristics could be used as a covariate. This statistical analysis is likely the most straightforward approach to the problem. However, the covariate approach may not always be possible. For instance, interactions with other experimental or statistical factors may prevent a direct comparison of target treatments, and thus, they may not be analyzed in the same statistical model. In this situation, another approach is possible; however, it is dependent on the presence of other treatments containing the same target and thus is not applicable to all target–neighbor studies.

In order to account for the biased neighbor treatment in our empirical study (Fig 2), the biomass of each of the targets was compared to their biomass measured on those same treatments in the previous year, when the neighbor treatments were not significantly different from one another. From this comparison, it could be seen that the biomass of target 2 was similar in both years, but that the biomass of target 1 had considerably dropped between year 1 and year 2. Although there was certainly a relationship between target biomass and neighbor biomass, it could not be definitively determined that the neighbors were larger because of an underperforming target or that the target was smaller due to some other factor causing those neighbors to increase in size. If the latter case is true, then target A would be expected to be larger and the neighbors not grown as much as they did. This means that target A means are below what they should have been and the neighbor treatments were consistent between target A and target B. This comparison gives some insight into the cause of the varied neighbor treatments, but it was not clear how much larger the target A means would have been in a consistent neighbor treatment.

Our next step depended on a separate treatment that was also part of the empirical study, where the target was grown alone (solo) without competitors. This solo treatment provided an upper bound for how much biomass the target A treatment could produce with no competition from neighbors at all, and the solo treatment had the largest mean biomass of any treatment. However, as the solo treatment biomass was still not significantly different from any of the other target treatments, we were confident that even if target A had grown larger when grown with the neighbors, the increased biomass would not lead to any changes in the significance of those target treatments. Thus, we were confident in our original interpretation of the target treatment results.

## Target–Neighbor Design – Going Forward

The typically unreported neighbor competitive response is likely due to the aforementioned narrower focus on target and neighbor experimental result. Also, it is likely that neighbor competitive effects may only be of concern a posteriori when a difference is readily apparent to the researcher and not a priori, which we suggest is the preferable approach. Alternatively, neighbor competitive response may be analyzed more frequently, but nonsignificant results often remain unreported in the literature. Regardless of whether a significant difference in neighbor competitive response is expected, confirmation should not be disregarded, as the omission of neighbor competitive response causes species interactions to be only partially quantified.

We advocate that future applications of the target–neighbor experimental design consider both the competitive effects and responses of targets and neighbors in order to provide a more complete understanding of the dynamics of plant competition. The analyses and controls in any experiment depend on the questions of interest, but it is important to take into account the interactions of each of the species or species mixtures. In addition to providing insight into target–neighbor interactions and guidance on analysis, information on neighbor competitive response can also be used to inform future decisions on experimental planting distance and relative plant positions. Measurement and analysis of neighbor competitive response should be performed to ascertain the effects of competition on all species involved in a target–neighbor or similarly constructed experimental designs.

## Conflict of Interest

None declared.

## Supporting information


**Appendix S1.** Resource 1.
