# Supplementary material for: The importance of analyzing neighbor competitive response in the target–neighbor experimental design
Source: Ecol Evol. 2015 Oct 26;5(22):5265–71. doi: 10.1002/ece3.1689 (PMC6102521; doi:10.1002/ece3.1689)
Supplement: Supplementary file 1 — Appendix S1. Resource 1. [file ECE3-5-5265-s001.docx]

**Online Resource 1**

Manuscript for: Ecology and Evolution

Title: The Importance of Analyzing Neighbor Competitive Response in the Target-Neighbor Experimental Design

Kevin J. Barry^12^, Michele R. Dudash^1^

^1^Department of Biology, University of Maryland College Park, MD 20742

^2^Department of Biology, West Virginia University Morgantown, WV 26506

Author for correspondence: kevin.barry@mail.wvu.edu, 412-848-7426

**Survey results of studies utilizing the target-neighbor design**

Articles found during a literature search of ISI Web of Science using the search terms “target neighbor” or “target neighbour” (American and British spellings, respectively) and “plant,” with no restriction on year through June 2014. From these results we selected manipulative studies where targets were deliberately planted into a neighbor community consisting of at least two neighbors. Results are listed in chronological order. Column headings and entries are as follows:

**Comm**: Was neighbor community planted as part of the experiment or was the target planted into a natural or unstructured community? (planted/natural)

**Type:** Did experiment involve separation of above-ground and below-ground competition or not? (a-b/no)

**Order**: Were neighbors established before the target, or were targets and neighbors planted together? (neighbors/same)

**NCE**: Was neighbor competitive effect or target competitive response (both are measures of neighbor influence on the target) reported? (yes/no)

**T#:** Was there more than one target treatment per neighbor treatment? (yes/no)

**NCR**: Was neighbor competitive response to targets reported (yes/no)

| **Author (year)** | **Comm.** | **Type** | **Order** | **NCE** | **T#** | **NCR** |
| --- | --- | --- | --- | --- | --- | --- |
| Clements (1927) | planted | a-b | neighbors | yes | no | no |
| Welbank (1961) | planted | a-b | neighbors | yes | no | - |
| Cook and Ratcliff (1984) | natural | a-b | neighbors - | | - | - |
| Cook and Ratcliff (1985) | natural | a-b | neighbors - | | - | - |
| Goldberg and Fleetwood (1987) | planted | no | neighbors | yes | yes | no |
| Reichenberger and Pyke (1990) | natural | a-b | neighbors - | | - | - |
| Goldberg and Landa (1991) | planted | no | together | yes | yes | no |
| Denslow et al. (1991) | natural | a-b | neighbors - | | - | - |
| Gill and Marks (1991) | natural | a-b | neighbors - | | - | - |
| Wilson and Tilman (1991) | natural | a-b | neighbors - | | - | - |
| Marvel et al. (1992) | planted | a-b | together | yes | no | - |
| Perera et al. (1992) | planted | a-b | together | yes | no | - |
| Putz and Canham (1992) | natural | a-b | neighbors - | | - | - |
| Seager et al. (1992) | natural | a-b | neighbors - | | - | - |
| Hartnett et al. (1993) | planted | no | together | yes | yes | no |
| Panetta and Randall (1993) | planted | no | together | yes | yes | no |
| Tremmel and Bazzaz (1993) | planted | no | together | yes | yes | no |
| Wilson (1993a) | natural | a-b | neighbors - | | - | - |
| Wilson (1993b) | natural | a-b | neighbors - | | - | - |
| Wilson and Tilman (1993) | natural | a-b | neighbors - | | - | - |
| Lindquist et al. (1994) | planted | no | together | yes | no | - |
| Thijs et al. (1994) | planted | no | together | yes | no | - |
| Dillenberg et al. (1995) | planted | a-b | together | yes | no | - |
| DiTomasso and Watson (1995) | planted | no | together | yes | no | - |
| Tremmel and Bazzaz (1995) | planted | no | together | yes | yes | no |
| Belcher et al. (1995) | natural | a-b | neighbors - | | - | - |
| Gerry and Wilson (1995) | natural | a-b | neighbors - | | - | - |
| Wilson and Tilman (1995) | natural | a-b | neighbors | - | - | - |
| Gibson and Skeel (1996) | planted | no | together | yes | no | - |
| Moora and Zobel (1996) | planted | no | together | yes | no | - |
| Twolan-Strutt and Keddy (1996) | natural | a-b | neighbors - | | - | - |
| van Auken and Bush (1997) | natural | a-b | neighbors - | | - | - |
| Vila (1997) | natural | a-b | neighbors - | | - | - |
| Peltzer et al. (1998) | natural | a-b | neighbors - | | - | - |
| Leishman (1999) | planted | no | neighbors | yes | yes | no |
| Cahill (2003) | natural | a-b | neighbors | - | - | - |
| Cahill and Casper (2000) | natural | a-b | neighbors | - | - | - |
| Humhprey and Pyke (2001) | planted | no | together | yes | no | - |
| Howard (2001) | natural | no | neighbors - | | - | - |
| Ronsheim and Anderson (2001) | planted | no | together | no | no | - |
| Cahill (2002) | natural | a-b | neighbors | - | - | - |
| Keddy et al.(2002) | planted | no | neighbors | yes | no | - |
| Weigelt et al. (2002) | planted | no | together | yes | yes | no |
| Franks (2003) | planted | no | together | yes | yes | no |
| Cheplick and Kane (2004) | planted | no | together | yes | yes | yes |
| Osunkoya et al. (2005) | planted | no | together | yes | yes | no |
| Weigelt et al. (2005) | planted | a-b | together | yes | yes | no |
| Harbur and Owen (2006) | planted | no | together | yes | no | - |
| Jankju-Borzelabad and Griffiths (2006) | planted | no | neighbors | yes | no | - |
| Ramseier and Weiner (2006) | planted | no | together | yes | no | - |
| James and Richards (2007) | planted | no | together | yes | yes | no |
| Rajaniemi (2007) | planted | no | together | yes | no | - |
| Weigelt et al. (2007) | planted | no | together | yes | yes | no |
| Hwang and Lauenroth (2008) | planted | no | together | yes | yes | no |
| Pollnac et al. (2008) | planted | no | together | yes | yes | no |
| Schmidt et al. (2008) | planted | no | together | yes | yes | no |
| Daneshgar and Jose (2009) | planted | no | neighbors | yes | no | - |
| Collins et al. (2010) | planted | no | together | yes | yes | no |
| Jiang et al. (2010) | planted | no | together | yes | no | - |
| Luo et al. (2010) | planted | no | together | yes | yes | no |
| Song et al. (2010) | planted | no | together | yes | yes | no |
| West et al. (2010) | planted | no | together | yes | no | - |
| Farrer and Goldberg (2011) | planted | no | together | yes | yes | no |
| Nagashima and Hikosaka (2011) | planted | a-b | together | yes | yes | no |
| Dyer et al. (2012) | planted | no | together | yes | no | no |
| Zhang and Lamb (2012) | planted | no | together | yes | yes | no |
| Peterson et al (2013) | planted | no | together | yes | no | no |
| Yang et al. (2013) | planted | a-b | together | yes | no | no |
| Xue et al. (2013) | planted | no | together | yes | yes | no |
| Gruntman et al. (2014) | planted | no | together | yes | no | no |
| Li et al. (2014) | planted | no | together | yes | yes | no |

**Literature survey citations**

Belcher, J.W., Keddy, P.A. & Twolan-Strutt, L. (1995) Root and shoot competition intensity

along a soil depth gradient. *Journal of Ecology* 83:673-682

Cahill, J.F. (2002) Interactions between root and shoot competition vary among species. *Oikos*

99:101-112

Cahill, J.F. (2003) Lack of relationship between below-ground competition and allocation to

roots in 10 grassland species. *Journal of Ecology* 91:532-540

Cahill, J.F. & Casper, B.B. (2000) Investigating the relationship between neighbor root biomass

and belowground competition: field evidence for symmetric competition belowground.

*Oikos* 90:311-320

Cheplick, G.P. & Kane, K.H. (2004) Genetic relatedness and competition in *Triplasis purpurea*

(Poaceae): Resource partitioning or kin selection? *International Journal of Plant Sciences*

165:623-630

Clements, F.E., Weaver, J.E. & Hanson, H.C. (1929) *Plant competition*. Carnegie Institution of

Washington,Washington

Collins, A., Hart, E.M. & Molofsky, J. (2010) Differential response to frequency-dependent

interactions: an experimental test using genotypes of an invasive grass. *Oecologia* 164:959-969

Cook, S. & Ratcliff, D. (1984) A study of the effects of root and shoot competition on the growth

of green panic (*Panicum maximum* var. *trichoglume*) seedlings in an existing grassland

using root exclusion tubes. *Journal of Applied Ecology* 21:971-982

Cook, S.J. & Ratcliff, D. (1985) Effect of fertilizer, root and shoot competition on the growth of

siratro (*Macroptilium* *atropurpureum*) and green panic (*Panicum maximum* var. *trichoglume*) seedlings in a native speargrass (*Heteropogon contortus*) sward. *Australian Journal of Agricultural Research* 36:233-245

Daneshgar P. & Jose, S. (2009) Role of species identity in plant invasions: experimental test

using *Imperata cylindrica*. *Biological Invasions* 11:1431-1440

Denslow, J.S., Newell, E. & Ellison, A.M. (1991) The effect of understory palms and cyclanths

on the growth and survival of Inga seedlings. *Biotropica* 23:225-234

Dillenburg, L., Whigham, D., Teramura, A. & Forseth, I. (1993) Effects of below-and

aboveground competition from the vines *Lonicera japonica* and *Parthenocissus*

*quinquefolia* on the growth of the tree host *Liquidambar styraciflua*. *Oecologia* 93:48-54

Ditommaso, A. & Watson, A.K. (1995) Impact of a fungal pathogen, *Colletotrichum coccodes*

on growth and competitive ability of *Abutilon theophrasti*. *New Phytologist* 131:51-60

Dyer, A.R., Hardison, J.L. & Rice, K.J. (2012) Phenology constrains opportunistic growth

response in *Bromus tectorum* L. *Plant Ecology* 213:103-112

Farrer, E.C. & Goldberg, D.E. (2011) Patterns and mechanisms of conspecific and heterospecific

interactions in a dry perennial grassland. *Journal of Ecology* 99:265–276

Franks, S.J. (2003) Competitive and facilitative interactions within and between two species of

coastal dune perennials. *Botany* 81:330-337

Gerry, A.K. & Wilson, S.D. (1995) The influence of initial size on the competitive responses of

six plant species. *Ecology* 76:272-279

Gibson, D. & Skeel, V. (1996) Effects of competition on photosynthetic rate and stomatal

conductance of *Sorghastrum nutans*. *Photosynthetica* 32:503-512

Gill, D.S. & Marks, P.L. (1991) Tree and shrub seedling colonization of old fields in central New

York. *Ecological Monographs* 61:183-205

Goldberg, D.E. & Fleetwood, L. (1987) Competitive effect and response in four annual plants.

*Journal of Ecology* 75:1131-1143

Goldberg, D.E. & Landa, K. (1991) Competitive effect and response: hierarchies and correlated

traits in the early stages of competition. *The Journal of Ecology* 79:1013-1030

Gruntman, M., Pehl, A.K., Joshi, S. & Tielbörger, K. (2014) Competitive dominance of the

invasive plant *Impatiens glandulifera*: using competitive effect and response with a

vigorous neighbour. *Biological Invasions* 16:141–151.

Harbur, M.M. & Owen, M.D.K. (2006) Influence of relative time of emergence on nitrogen

responses of corn and velvetleaf. *Weed Science* 54:917-922

Hartnett, D., Hetrick, B., Wilson, G. & Gibson, D. (1993) Mycorrhizal influence on intra-and

interspecific neighbour interactions among co-occurring prairie grasses. *Journal of*

*Ecology* 81:787-795

Howard, T.G. (2001) The relationship of total and per-gram rankings in competitive effect to the

natural abundance of herbaceous perennials. *Journal of Ecology* 89: 110-117

Humphrey, L.D. & Pyke, D.A. (2001) Ramet spacing of *Elymus lanceolatus* (thickspike

wheatgrass) in response to neighbour density. *Canadian Journal of Botany* 79:1122-1126

Hwang, B. & Lauenroth, W. (2008) Effect of nitrogen, water and neighbor density on the growth

of *Hesperis matronalis* and two native perennials. *Biological Invasions* 10:771-779

James, J.J. & Richards, J.H. (2007) Influence of temporal heterogeneity in nitrogen supply on

competitive interactions in a desert shrub community. *Oecologia* 152:721-727

Jankju-Borzelabad, M. & Griffiths, H. (2006) Competition for pulsed resources: an experimental

study of establishment and coexistence for an arid-land grass. *Oecologia* 148:555-563

Jiang, J., Kong, F., Gu, X., Chen, K., Zhao, S. & Wang, J. (2010) Influence of intraspecific

interaction and substrate type on initial growth and establishment of *Hydrilla verticillata*.

*Hydrobiologia* 649:255-265

Keddy, P., Nielsen, K., Weiher, E. & Lawson, R (2002) Relative competitive performance of 63

species of terrestrial herbaceous plants. *Journal of Vegetation* 13:5-16

Leishman, M.R. (1999) How well do plant traits correlate with establishment ability? Evidence

from a study of 16 calcareous grassland species. *New Phytologist* 141:487-496

Li, F., Xie, Y., Liu, Y., Tang, Y., Chen, X., Deng, Z., Hu, J. & Liu, N. (2014) Negative influence

of burial stress on plant growth was ameliorated by increased plant density in *Polygonum*

*hydropiper*. Limnologica 45:33-37

Lindquist, J., Rhode, D., Puettmann, K. & Maxwell, B. (1994) The influence of plant population

spatial arrangement on individual plant yield. *Ecological Applications* 4:518-524

Luo, W., Xie, Y., Chen, X., Li, F. & Qin, X. (2010) Competition and facilitation in three marsh

plants in response to a water-level gradient. *Wetlands* 30:525-530

Marvel, J.N., Beyrouty, C.A. & Gbur, E.E. (1992) Response of soybean growth to root and

canopy competition. *Crop Science* 32:797-801

Moora, M. & Zobel, M. (1996) Effect of arbuscular mycorrhiza on inter-and intraspecific

competition of two grassland species. *Oecologia* 108:79-84

Nagashima, H. & Hikosaka, K. (2011) Plants in a crowded stand regulate their height growth so

as to maintain similar heights to neighbours even when they have potential advantages in

height growth. *Annals of Botany* 108:207-214.

Osunkoya, O.O., Othman, F.E. & Kahar, R.S. (2005) Growth and competition between seedlings

of an invasive plantation tree, *Acacia mangium*, and those of a native Borneo heath-forest

species, *Melastoma beccarianum*. *Ecological Research* 20:205-214

Panetta, F. & Randall, R. (1993) *Emex australis* and the competitive hierarchy of a grazed annual

pasture. *Journal of Applied Ecology* 30:373-379

Peltzer, D.A., Wilson, S.D. & Gerry, A.K. (1998) Competition intensity along a productivity

gradient in a low-diversity grassland. *American Naturalist* 151:465-476

Perera, K.K., Ayres, P.G., and Gunasena, H.P.M. (1992) Root growth and the relative

importance of root and shoot competition in interactions between rice (*Oryza sativa*) and

*Echinochloa crus-galli*. *Weed Research* 32:67-76

Peterson, M.L., Rice, K.J., Sexton, J.P. (2013) Niche partitioning between close relatives

suggests trade-offs between adaptation to local environments and competition. *Ecology*

*and Evolution* 3:512–522

Pollnac, F., Maxwell, B. & Menalled, F. (2009) Weed community characteristics and crop

performance: a neighbourhood approach. *Weed Research* 49:242–250

Putz, F.E. & Canham, C.D. (1992) Mechanisms of arrested succession in shrublands: root and

shoot competition between shrubs and tree seedlings. *Forest Ecology and Management*

49:267-275

Rajaniemi, T.K. (2007) Root foraging traits and competitive ability in heterogeneous soils.

*Oecologia* 153:145-152.

Ramseier, D. & Weiner, J. (2006) Competitive effect is a linear function of neighbour biomass in

experimental populations of *Kochia scoparia*. *Journal of Ecology* 94:305-309

Reichenberger, G. & Pyke, D.A. (1990) Impact of early root competition on fitness components

of four semiarid species. *Oecologia* 85:159-166

Ronsheim, M.L. & Anderson, S.E. (2001) Population-level specificity in the plant-mycorrhizae

association alters intraspecific interactions among neighboring plants. *Oecologia*

128:77-84

Schmidt, C.D., Hickman, K.R., Channell, R., Harmoney, K., & Stark, W. (2008) Competitive

abilities of native grasses and non-native (*Bothriochloa* spp.) grasses. *Plant Ecology*

197:69-80

Seager, N.G., Kemp, P.D. & Chu, A.C.P. (1992) Effect of root and shoot competition from

established hill-country pasture on perennial ryegrass. *New Zealand Journal of Agricultural Research* 35:359-363

Song, L., Zhang, D., Li, F., Fan, X., Ma, Q. & Turner, N. (2010) Soil water availability alters the

inter-and intra-cultivar competition of three spring wheat cultivars bred in different eras. *Journal of Agronomy and Crop Science* 196:332-335

Thijs, H., Shann, J. & Weidenhamer, J. (1994) The effect of phytotoxins on competitive outcome

in a model system. *Ecology* 75:1959-1964

Tremmel, D. & Bazzaz, F. (1993) How neighbor canopy architecture affects target plant

performance. *Ecology* 74:2114-2124

Tremmel, D. & Bazzaz, F. (1995) Plant architecture and allocation in different neighborhoods:

implications for competitive success. *Ecology* 76:262-271

Twolan-Strutt, L. & Keddy, P.A. (1996) Above-and belowground competition intensity in two

contrasting wetland plant communities. *Ecology* 77:259-270

Van Auken, O. & Bush, J. (1997) Growth of *Prosopis glandulosa* in response to changes in

aboveground and belowground interference. *Ecology* 78:1222-1229

Vilá, M. (1997) Effect of root competition and shading on resprouting dynamics of *Erica*

*multiflora* L. *Journal of Vegetation Science* 8:71-80

Weigelt, A., Steinlein, T. & Beyschlag W (2002) Does plant competition intensity rather depend

on biomass or on species identity? *Basic and Applied Ecology* 3:85-94.

Weigelt, A., Schumacher, J., Walther, T., Bartelheimer, M., Steinlein, T. & Beyschlag, W.

(2007) Identifying mechanisms of competition in multi-species communities. *Journal of Ecology* 95:53-64.

Weigelt, A., Steinlein, T. & Beyschlag, W. (2005) Competition among three dune species: the

impact of water availability on below-ground processes. *Plant Ecology* 176:57-68

West, A.M., Richardson, R.J., Arellano, C. & Burton, M.G. (2010) Bushkiller (*Cayratia*

*japonica*) growth in interspecific and intraspecific competition. *Weed Science*

58:195-198.

Wilson, S.D. (1993a) Competition and resource availability in heath and grassland in the Snowy

Mountains of Australia. *Journal of Ecology* 81: 445-451

Wilson, S.D. (1993b) Belowground competition in forest and prairie. *Oikos* 68: 146-150

Wilson, S.D. & Tilman, D. (1991) Components of plant competition along an experimental

gradient of nitrogen availability. *Ecology* 72:1050-1065

Wilson, S.D. & Tilman, D. (1993) Plant competition and resource availability in response to

disturbance and fertilization. *Ecology* 74:599-611

Wilson, S.D. & Tilman, D. (1995) Competitive responses of eight old-field plant species in four

environments. *Ecology* 76:1169-1180

Xue, W., Huang, L., Dong, B.C., Zhang, M.X. & Yu, F.H. (2013) Patchy distributions of

competitors affect the growth of a clonal plant when the competitor density is high. PLoS

ONE 8(10): e78221. doi:10.1371/journal.pone.0078221

Yang, H., Yu, Z., Zhang, Q., Tang, J. & Chen, X. (2013) Plant neighbor effects mediated by

rhizosphere factors along a simulated aridity gradient. *Plant and Soil* 369: 165-176

Zhang, S. & Lamb, E.G. (2012) Plant competitive ability and the transitivity of competitive

hierarchies change with plant age. *Plant Ecology* 213:15-23
